# Implementation and Measurement of Shared Decision Making in Gynaecological Oncology Outpatient Setting at a Tertiary Cancer Centre

**DOI:** 10.3390/cancers17193168

**Published:** 2025-09-29

**Authors:** Sarah Ahmed, Benitta Mathews, David Griffiths, Yvonne Anderson, Nithya Ratnavelu, Tineke Vergeldt

**Affiliations:** 1 Northern Gynaecological Oncology Centre, Queen Elizabeth Hospital, Gateshead Health NHS Foundation Trust, Gateshead NE9 6SX, UK; sarah.ahmed@uhbw.nhs.uk (S.A.); benitta.mathews@nhs.net (B.M.); david.griffiths6@nhs.net (D.G.); yvonne.anderson6@nhs.net (Y.A.); nithya.ratnavelu@nhs.net (N.R.); 2 Department of Gynaecological Oncology Surgery, St Michael’s and Bristol Royal Infirmary Hospitals, University Hospitals Bristol and Weston NHS Foundation Trust, Bristol BS2 8EG, UK

**Keywords:** gynaecological oncology, shared decision-making, BRAN tool, SDM Q-9 questionnaire, quality improvement

## Abstract

**Background**: Shared Decision-Making (SDM) is important for patient-centred care, especially in areas such as gynaecological oncology where treatment decisions are often multifaceted. This study aimed to implement and measure SDM in a gynaecological oncology outpatient clinic, specifically assessing the impact of the BRAN (Benefits, Risks, Alternatives, Nothing) tool on patient and physician perceptions. **Methods**: A two-phased prospective observational and survey mixed-methodology study was conducted at the tertiary Northern Gynaecological Oncology Centre (NGOC) outpatient clinic in Gateshead, United Kingdom, from October 2023 to November 2024. SDM champions provided staff training. Patient and physician perspectives were measured using the nine-item Shared Decision Making Questionnaire (SDM-Q-9). Phase one was a baseline assessment; phase two involved the implementation of BRAN posters and leaflets. Statistical analyses included the Mann–Whitney U Test and Fisher’s Exact Test. A post-implementation online staff survey was conducted. **Results**: A total of 207 patients and 13 physicians participated: 107 patients and 13 physicians in phase one and 100 patients and 12 physicians in phase two. Whilst no statistically significant difference in patients’ perceptions of SDM was found between phases (*p* = 0.73), physicians’ perceptions showed a statistically significant improvement after BRAN tool implementation (*p* < 0.01). The staff survey results indicated that 84% observed increased patient involvement, and 92% agreed that SDM helped achieve consultation goals. **Conclusions**: The implementation of SDM at the NGOC led to a statistically significant improvement in the subjective use of SDM by physicians’, despite no significant change in patients’ perceptions, possibly due to high baseline levels. Staff reported increased patient engagement and improved consultation styles. These findings support implementing SDM in gynaecological oncology outpatient settings

## Simple Summary

This study looked at how doctors and patients can work together to make treatment decisions, particularly in gynaecological cancer outpatient clinics. The study aimed to see whether introducing a simple tool, called BRAN, could improve these shared discussions in a clinic. The findings showed that doctors felt they were better at involving patients after using the tool, and staff noticed patients becoming more engaged in their care. This work highlights the importance of open communication in healthcare and offers insights for other medical fields looking to empower patients in their treatment choices, ultimately leading to greater patient satisfaction and quality of life.

## 1. Introduction

Shared Decision-Making (SDM) is a process in which patients and physicians work together to reach a treatment decision that considers patients’ preferences and goals and the best available evidence [1]. It is an essential aspect of patient-centred care, which is defined as ‘care that respects and responds to individual patient needs, values, and preferences, ensuring that the patient values guide clinical decisions’ [2]. The United Kingdom (UK) Health and Social Care Act advocates for individual involvement in decisions about their care. The National Institute for Health and Care Excellence (NICE) has provided guidelines on utilising SDM, and the General Medical Council (GMC) supports its implementation [3].

Effective SDM communication improves treatment adherence, survival rates, and quality of life for patients with cancer [4]. To implement SDM, six steps during consultation have been suggested: (1) Recognise that a decision needs to be made. (2) Explain the need for participation. (3) Present all reasonable treatment options including the benefits and risks of each. (4) Understand what is important to the patient in making this decision. (5) Collaborate with the patient to choose the option that best aligns with their values and preferences. (6) Implement the decision into practice [5]. SDM tools and decision aids can be used to help implementation. One such tool is the Choosing Wisely initiative’s BRAN (Benefits, Risks, Alternatives, Nothing) tool. This SDM tool aids patient involvement in decision-making and encourages patients to consider four key elements: the benefits of the treatment or choice, the associated risks, alternatives, and the option of doing nothing (BRAN pneumonic) [6]. Randomised controlled trials (RCTs) have shown that decision aids enhance patients’ involvement in decision-making, improve the quality and efficiency of decisions, and reduce decisional conflict [7]. Alongside decision aids, healthcare provider training was an important effective factor that aids the adoption and implementation of SDM. This was concluded in a Cochrane review that analysed 5 RCTs [8,9,10,11,12] and showed that the implementation of SDM is multifaceted [13].

The SDM process during gynaecological cancer treatment consultations is complex. This complexity is due to several factors, including the course of the disease, the patient-provider relationship, and the lack of comprehensive evidence regarding all available treatment options. This is especially the case when a patient opts for conservative management or no treatment at all, e.g., because of fertility-sparing intent. A systematic review examining the experiences of cancer patients of reproductive age in making fertility decisions revealed that they often experienced decisional conflict. Physicians’ active participation was essential, and they recommended using patient decision aids to facilitate the decision-making process [14].

Another scenario where opting for a non-standard treatment can be a valid choice is in older patients and those who are frail and have multiple comorbidities. The application and measurement of SDM are particularly important here, because of the lack of evidence on long-term survival and functional status [15]. A study suggested that gynaecological oncology surgeons may be more likely to recommend surgery for older women with endometrial cancer compared to other healthcare providers [16]. However, some women in their late 80s declined surgery during the study. The study authors proposed offering standard endometrial cancer treatment to fit older patients while recommending more in-depth risk discussions for frail older patients.

The journey of women with gynaecological cancer often involves various treatment modalities, such as chemotherapy, surgery, hormone therapy, and radiotherapy, coordinated by different specialists working in separate departments or hospitals [17]. Therefore, involving women in their treatment decisions is crucial both before initiating treatment and while establishing treatment goals, as their preferences may vary and change during the treatment pathway [17]. Currently, there is minimal evidence on the application of SDM in gynaecological oncology, and studies have suggested a high demand for SDM due to the insufficient evidence on the information needs of different cancer patients [18].

Within the UK, the Commissioning for Quality and Innovation (CQUIN) group encourages organisations to train physicians in implementing SDM and to use validated tools for evaluations [19]. Through collaboration with CQUIN, the authors proposed to implement SDM into the gynaecological oncology surgery outpatient consultation at a tertiary cancer centre. The aim of this study was to measure SDM at baseline and after implementation of the BRAN tool to identify areas for improvement in the consultation process.

## 2. Materials and Methods

A two-phased prospective observational and survey mixed-methodology clinical study was conducted in the Northern Gynaecological Oncology Centre (NGOC) outpatient department in Gateshead, UK, between October 2023 and November 2024. The NGOC is the tertiary referral centre for surgical treatment of gynaecological cancers in the North East and North Cumbria, covering an area with a population of 4 million. This study was defined as a quality improvement project according to the UK Policy Framework for Health and Social Care Research, as it did not alter the patient pathway or treatment [20]. The decision tool ‘Is my study research?’ from the UK Policy Framework for Health and Social Care Research was used to verify this [21]. According to the glossary in this tool service evaluation is designed and conducted to define or judge current care and may include questionnaires for analysis. Service development or improvement seeks to find out what improvement can be achieved within that service, and this may involve a new intervention or service. Service evaluation, improvement and development work does not require review by an NHS Research Ethics Committee [20,21]. The local research and development department at the Gateshead Health NHS Foundation Trust confirmed the study did not require approval by an Ethics committee.

A gynaecological oncology consultant, resident doctor, and cancer nurse specialist (CNS) were nominated as SDM champions. They completed theory-based e-learnings and webinars on SDM provided by NHS England, Skills for Health and the Personalised Care Institute, and organised teaching to physicians and staff nurses prior to the start of the study in September 2023. The teaching continued throughout the study phases as part of weekly departmental teaching.

Patient and physician perspectives on SDM were measured using the nine-item Shared Decision Making (SDM-Q-9) Questionnaire [22], which is the recommended tool to measure patient recorded SDM outcomes by the National Institute for Health and Care Excellence (NICE) [23]. This validated instrument was designed to evaluate the process of SDM during medical consultations and has been assessed as quick to complete and suitable for use in research [22]. In the questionnaire, each item outlines a distinct step in the SDM process. Participants rate each statement on a 6-point Likert scale, where 0 signifies ‘completely disagree that this step of SDM was taken by the physician’ and 5 indicates ‘completely agree that this step of SDM was taken by the physician’. The total score for each patient can range from 0 to 45, with higher scores showing a greater application of SDM [24]. The physician completed the physician’s version of the questionnaire using the same statements but from their perspective; ‘completely agree/disagree that I took this step of SDM during the consultation’. Patients anonymously completed the questionnaires, and neither patient nor physician knew the other’s responses. For each patient, only one physician completed the questionnaire on a one-to-one basis. The questionnaires were coded to match the patient’s and physician’s answers. Both patients and physicians were encouraged to fill out the questionnaires immediately after the consultation, although they were allowed to complete them at a more convenient time if needed.

The inclusion criteria were consecutive newly referred patients diagnosed at the gynaecological oncology outpatient clinic, as identified from clinic lists. A patient could only participate once as only new patients seen in clinic for their first appointment were eligible. All physicians seeing newly referred patients in the gynaecological oncology clinic participated. The physician decided whether to ask the patient to complete the questionnaire after the consultation. The consent was obtained verbally only, and patients who did not consent did not fill out the questionnaire. Phase one of the study was the baseline assessment, prior to implementation of the BRAN tool. Phase two was the post implementation phase. During phase two, patient involvement in SDM was encouraged by displaying BRAN posters in patients’ waiting areas and sending out BRAN leaflets to patients with their appointment letters. In case a patient did not receive the leaflet prior to the clinic appointment, it was given at clinic reception for them to read whilst waiting for a physician’s consultation. A power calculation was not performed as data on expected outcomes were lacking. As the CQUIN collaboration had set deadlines to provide updates on progression, the aim was to include approximately 100 patients in 6 months during phase one, followed by approximately 100 patients in 6 months during phase two.

Visual inspection of histograms indicated that the data were not normally distributed, with a clear ceiling effect, as most responses clustered at the maximum score. This was further supported by the nature of the SDM-Q-9 Likert scale and the summed scores showing skewness towards higher values. Hence, statistical analyses were performed using appropriate non-parametric tests. The two-tailed Mann–Whitney U Test was used to compare total SDM-Q-9 scores between phase one and phase two for both patients and physicians. A *p*-value of <0.05 was considered statistically significant. Fisher’s Exact Test was used to analyse whether the proportion of patients and physicians scoring the maximum possible SDM-Q-9 score differed significantly between phases one and two. A *p*-value of <0.05 was considered statistically significant.

After phase two, to effectively gauge staff opinions during and after the implementation of SDM, an online survey was conducted anonymously. This survey was developed after literature review of similar studies on SDM implementation [6], but it was not tested. The survey was emailed through Microsoft Forms (Cloud service, Microsoft Corporation, Redmond WA, USA) to the NGOC consultants, specialty resident doctors, and CNSs who had been involved in the new patient clinics during this study. It included 8 statements with yes/no questions, 0 to 10 ratings, and open-ended questions. Data were collected from the 11 December 2024 to the 11 March 2025 and analysed using Microsoft Excel (version 2507 Microsoft Corporation, Redmond WA, USA).

## 3. Results

Questionnaires were collected between October 2023 and November 2024 during which 572 new patients were seen at the NGOC outpatient clinic. A total of 207 patients and 13 physicians participated: 107 patients and 13 physicians in phase one and 100 patients and 12 physicians in phase two. In Phase one, a total of 12 physicians participated, comprising 8 consultants and 4 registrars. In Phase two, 13 physicians participated, including 8 consultants and 5 registrars. There was an overlap of 8 consultants and 2 registrars between the two phases. The median patients’ ages were 63 years (range 32–88 years) in phase one and 65 years (range 18–89 years) in phase two. The distribution of the different cancer types in phase one and phase two is shown in Table 1.

Patients’ questionnaires were fully completed by 185 out of 207 patients (89.4%), and 197 out of 207 physicians’ questionnaires were fully completed (95.2%). The maximum score of 45 (indicating ideal SDM) was given in 74 out of 96 (77.1%) fully completed questionnaires by patients and 10 out of 99 (10.1%) fully completed questionnaires by physicians in phase one and by 65 out of 89 (73.0%) and 24 out of 98 (24.5%), respectively, in phase two. Fisher’s Exact statistics test showed no statistically significant difference in the proportion of maximum scores for patients between phase one and phase two (*p* = 0.61). However, for physicians, there was a statistically significant increase in the proportion of maximum scores after the implementation of the BRAN tools (*p* < 0.01).

**Figure 1:**
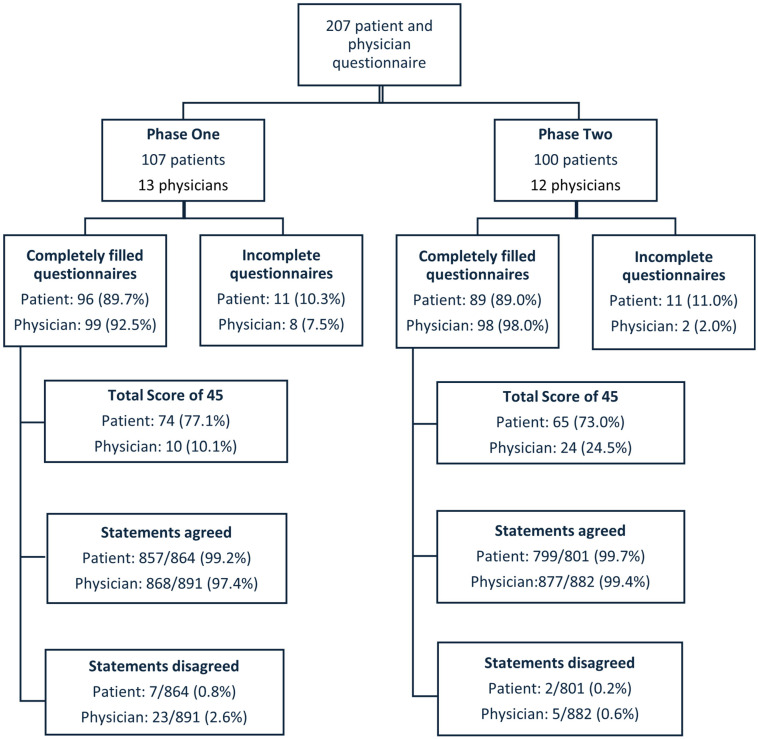
Phases one and two SDM-Q-9 questionnaires results.

Responses were categorised into two groups: scores of 0 to 2 were considered as ‘disagree this step of SDM was taken’, while scores of 3 to 5 were considered as ‘agree this step of SDM was taken’. Figure 1 shows the flowchart of these findings.

**Figure 2:**
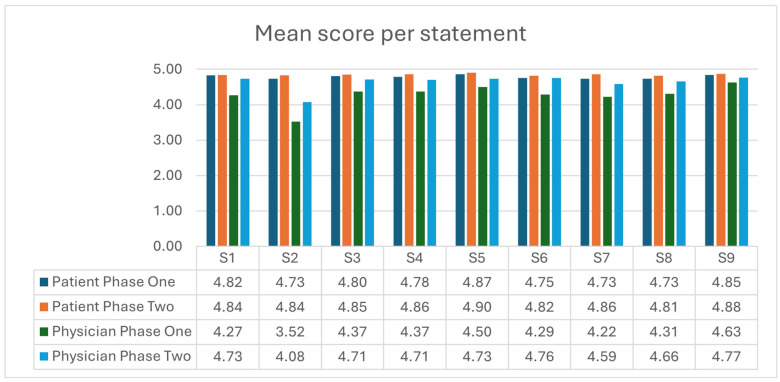
Mean scores per statement for patients and physicians in phase one and phase two.

Figure 2 illustrates the mean scores per statement for patients and physicians in both phase one and phase two. The mean total scores for fully completed questionnaires were 43.6 (range 29 to 45) for patients and 39.0 (range 25 to 45) for physicians in phase one, 43.7 (range 34 to 45) for patients, and 41.8 (range 33 to 45) for physicians in phase two. When comparing total scores using the two-tailed Mann–Whitney U Test, there was no statistically significant difference in patients’ perceptions of SDM between phase one and phase two (*p* = 0.73). In contrast, physicians’ perceptions showed a statistically significant improvement with a *p*-value of <0.01. There was a statistically significant difference between patients’ and physicians’ perceptions of SDM in both phase one and phase two (*p* < 0.001).

The most disagreement, defined as ‘0/completely disagree’, ‘1/strongly disagree’, or ‘2/somewhat disagree that this SDM step was taken by physician’, in the patient questionnaires was with statements 2 ‘My doctor wanted to know exactly how I want to be involved in making the decision’ and 8 ‘My doctor and I selected a treatment option together’. Among physicians, the most disagreement occurred with statement 2 in phase one, ‘I wanted to know exactly how the patient wanted to be involved in making the decision’, and with statement 3 in phase two: ‘I told my patient about different options for treating their condition’.

Thirteen staff members participated in a survey study to assess staff experience in the implementation of SDM in the department: four consultants (57% out of the seven consultants who filled out the SDM-Q-9 questionnaires during the study), seven specialty resident doctors (43%, i.e., 3 out of the seven who filled out the SDM-Q-9 questionnaires during the study) and two CNSs (who are present in clinic with the physician during the consultations). All consultants who completed the survey participated in both phases of the study. However, not all resident doctors who filled out the survey were involved in both phases. The survey was conducted anonymously, which means precise information on the number of participants who took part in both phases and details about the overlap at the resident doctor levels are not available. The survey results show that 84% of staff members observed increased patient involvement in decision-making, and 92% agreed that SDM approach helped them achieve their consultation goals, which included explaining the disease, options, and finalising decisions that honour patients’ wishes. The average perceived score for physicians’ SDM consultation style was 6 (range from 5 to 8) on a scale from 1 to 10 prior to implementation, and it increased to an average of 8 (range from 5 to 10) after implementation.

**Figure 3:**
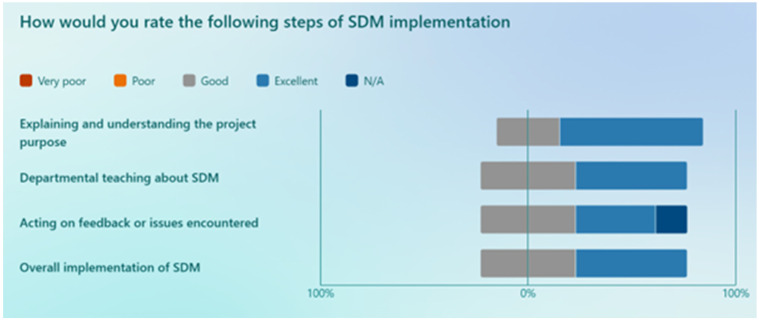
Staff rating of various steps of SDM implementation.

The results of the staff members’ rating of various steps in the implementation process are shown in Figure 3. Upon analysing data, the following challenges were reported: ‘remembering to fill out and give the questionnaires’, ‘not all patients wanted to be involved’, ‘some questions in the questionnaire were a bit hard to rate’, ‘consultations take longer’, ‘some of the phrases on the questionnaire didn’t reflect true practice, e.g., asking patients how would you like to be involved’. On average, the staff members who completed the post-study survey had a 76% likelihood of continuing with the newly adopted SDM style. Additionally, 90% of the staff members expressed a willingness to recommend the implementation of SDM to a colleague.

## 4. Discussion

SDM was successfully implemented and measured at the NGOC outpatient clinic, Gateshead, UK, through continuous education of staff and collaboration with CQUIN. The levels of subjective SDM based on the questionnaires were high, both at baseline and after implementation of BRAN. The staff survey shows a noticeable increase in patient engagement in treatment decision-making and changes in the physicians’ consultation style after SDM implementation. There was a statistically significant improvement in physicians’ subjective SDM consultation style after implementing SDM at the NGOC, with no significant difference in SDM following the implementation of the BRAN leaflet and poster from the patient’s perspective.

The lack of significant difference between patients’ scores before and after implementation of the BRAN tool may be attributed to the high levels of SDM rated by the patients at the baseline phase. In a systematic review assessing the use of the 9-item SDM questionnaires, four out of five studies found no significant differences in mean scores between the intervention and control groups. In a further study, the difference observed was minimal. This suggests that the SDM-Q-9 questionnaires may have limitations in their sensitivity to detect changes when it comes to patients’ perception of SDM [25].

The statistically significant improvement in mean total score and percentage of questionnaires with the maximum score of 45 from the physicians’ perspective may reflect their learning curve as they gained more experience with the newly adopted SDM process over time. This included utilising the questionnaires and participating in the SDM departmental teaching. In a study looking at learning SDM in clinical practice, it was concluded that residents’ skills in SDM were mostly developed with clinical practice and not during undergraduate medical education [26]. The improvements were specific to SDM skills, attitude, and knowledge. In this study, the difference or improvement could hypothetically also be explained by the fact that some physicians participated in phase one only or phase two only and were not involved in both phases.

Another point to consider is the distribution of patients by cancer types and their involvement in different phases of the study. Notably, the percentage of patients with vulval cancer is higher than expected in a gynaecological oncology population, which may have influenced the results. This may be attributed to the fact that the consultants who included a high number of patients in the study had a specific interest in vulval cancer, and one of these consultants was an SDM champion. When examining the distribution of cancer types between phase one and phase two, it is evident that phase one had a higher number of patients with ovarian cancer, whilst phase two had more patients with endometrial cancers. This variation in patient distribution and raised vulval cancer cases could have impacted the results, as managing ovarian cancer could involve more complex decision-making than vulval or endometrial cancers, given the multiple primary treatment options available. Currently it is unclear if the benefit of implementing SDM in gynaecological cancer consultations is evenly distributed amongst all different cancer types. Each cancer type has different treatment options and complexity in decision-making. A study that investigated the effect of involving ovarian cancer survivors in SDM showed better mental health survivorship, greater vitality, and better role-emotional health in survivorship. Literature on the other cancer types is limited [16,27,28].

In this study, it was found that a minority of patients disagreed with statements that physicians inquired about how patients wanted to participate in treatment decisions and collaborated with them on selecting treatment options. A study by Kane et al. suggested that it is important to recognise that patients and physicians may have significant differences in their perceptions of how much involvement patients would like and how they view the benefits of certain treatment options when making decisions [17]. In a study by Fitch et al. assessing women’s perspectives on treatment decision-making for ovarian cancer, most women stated that treatment decisions were primarily made by physicians [29]. They reported having minimal involvement in both initial treatment decisions and in adjuvant treatment decisions. Many women felt they were trapped in a series of events over which they had little control, encountered challenges when trying to participate in the decision-making process, and found it overwhelming to make decisions while coming to terms with the diagnosis of cancer [29]. All these studies, including the current one, emphasise the importance of discussing the level of involvement a patient desires at the start of the decision-making process. It may be beneficial for physicians to have access to their patients’ completed questionnaires to identify any discrepancies in the perceived extent of SDM during a particular consultation.

The findings of the staff survey at the end of the project show alignment with a Dutch survey amongst oncologists, where 95% agreed that patients should be involved in treatment decisions, and 73% preferred collaborating with patients when making decisions [17]. Another survey showed that oncologists are particularly interested in engaging in SDM with their patients [30]. However, it was indicated in a literature review that physicians’ medical expertise, values, personal beliefs, and communication styles affect the application of SDM [31]. Some physicians have perceived barriers to SDM. This includes, but is not limited to, insufficient training, time constraints, workload, the nature of the clinical situation, and patient characteristics that hinder SDM [17]. Some of these barriers were identified in the staff survey as challenges encountered during SDM implementation. The potential barriers and solutions to SDM barriers were suggested in the literature [32,33,34]. There are organisational, cultural, financial, and methodological barriers that reduce the proper implementation of SDM in clinical practices. Suggested solutions mentioned in the literature are promoting patients to accept responsibility to be involved in decision-making, a supportive policy for training of staff, recognition that SDM will lead to improved health outcome, and the use of simple patient-friendly language to target consultations with elderly and low health literacy patients.

The strength of this study lies in its prospective design and mixed methodology, combining patients’ and physicians’ validated questionnaires and survey elements. It was conducted over a time period of more than a year and included a decent number of patients and physicians. Limitations of the study are the lack of power calculation, which could have influenced the lack of significance in patients’ perspectives on SDM, and several potential sources of bias that need to be addressed. Firstly, not all eligible patients attending the clinic were included in the study. Of the 572 new patients seen at the NGOC outpatient clinic during the inclusion period, 207 patients (36.2%) were included. This is because it was left to the physicians to decide whether to consent the patient for inclusion or not, and some patients declined participation in the study. This may have excluded patients with language barriers, low literacy, learning difficulties, those who had more difficult consultations, or perhaps those where the physicians were not satisfied with their consultation style. This could have influenced the outcomes. Two staff members reported in the survey that not all patients wanted to be involved in the study, but the numbers of patients invited to participate, how many declined participation, and their reasons for declining, were not collected during this study for practical reasons.

Another limitation is that most patients completed the questionnaire immediately after their consultation, which may have influenced their responses due to unrelated factors, such as stress and anxiety about their cancer diagnosis and treatment. Some patients were scheduled for appointments at short notice by phone, without receiving an appointment letter including the BRAN leaflet before their consultation. To address this, BRAN leaflets were distributed to patients at the clinic reception. While all patients used the same waiting area in the outpatient clinic where the BRAN posters were displayed, there is insufficient information regarding their exposure to the BRAN tool. No survey was conducted amongst patients to assess their exposure to and perception of the BRAN tool or their desire to be involved in decision-making. Another point of bias was that the physician who recruited the highest number of patients was also an SDM champion who did the e-learning and webinars at the start of the study and provided teaching on SDM.

## 5. Conclusions

In summary, implementing SDM at the NGOC led to a statistically significant improvement in the subjective use of SDM by physicians. There was no significant difference in the patients’ perception of SDM before and after implementing the BRAN tool. This may be due to the high baseline level of perceived SDM by the patients. Despite this, staff members reported increased patient engagement in treatment decisions and improvements in the physicians’ consultation style. These findings support the recommendation to implement SDM in clinical practice in a gynaecological oncology outpatient setting.

A Cochrane review and meta-analysis on interventions that increase the use of SDM concluded that there is uncertainty regarding the effectiveness of these interventions because the certainty of evidence is low or very low [35]. To further improve the SDM process in gynaecological oncology, more evidence is required. Focus groups would be a useful tool to understand patient preferences in communication styles and their level of desired involvement in decision-making. To address a discrepancy in perception of SDM between physicians and patients, unblinding physicians to their patients’ perceived level of SDM after the consultation and providing a real-time reflection can be helpful. Additionally, developing decision aids in gynaecological oncology can enhance patients’ understanding of their treatment choices and improve SDM and ultimately patient satisfaction and quality of life.

## Figures and Tables

**Table 1:** Cancer type demographics.

Cancer Type	Phase One		Phase Two	
	Number of patients (%)	Mean Age (Range)	Number of patients (%)	Mean Age (Range)
Ovarian cancer	42 (39%)	63 (34–83)	20 (20%)	63 (18–89)
Isolated pelvic mass	16 (15%)	64 (52–76)	21 (21%)	60 (35–86)
Endometrial cancer	14 (13%)	69 (56–80)	30 (30%)	68 (55–87)
Vulval cancer	23 (21%)	64 (37–88)	17 (17%)	70 (34–89
Cervical cancer	5 (5%)	42 (32–50	9 (9%)	66 (37–88)
Other	7 (7%)	75 (55–83)	3 (3%)	61 (51–78)

## Data Availability

Data will be available upon reasonable request.

## Abbreviations

The following abbreviations are used in this manuscript:

SDM	Shared Decision-Making
UK	United Kingdom
NICE	National Institute for Health and Care Excellence (NICE)
GMC	General Medical Council
BRAN	Benefits, Risk, Alternatives, Nothing
CQUIN	Commissioning for Quality and Innovation
NGOC	Northern Gynaecological Oncology Centre
CNS	Clinical Nurse Specialist
